# Education Research: Case-Based and Near-Peer Teaching on Functional Neurologic Disorder in the Neurology Clerkship: A Cross-Site Comparative Pilot Study

**DOI:** 10.1212/NE9.0000000000200342

**Published:** 2026-07-02

**Authors:** Martin Viola, Haatem Reda, Alexandra Hovaguimian, Jonah Zuflacht

**Affiliations:** 1 Harvard Medical School, Boston, MA;; 2 Department of Neurology, Massachusetts General Hospital, Boston, MA; and; 3 Department of Neurology, Beth Israel Deaconess Medical Center, Boston, MA.

## Abstract

**Background and Objectives:**

Undergraduate medical education on functional neurologic disorders (FNDs) is limited and misconceptions regarding the diagnosis of FND are common, negatively affecting medical student knowledge, and attitudes about FND. We developed a multicomponent educational program on FND and delivered it to medical students previously assigned at random to 1 of 2 neurology clerkship sites, evaluating its effects on FND knowledge and attitudes.

**Methods:**

This program, run monthly from January to December 2025, consisted of a case-based session delivered by an attending neurologist followed by a near-peer session delivered by a medical student. Primary outcome was FND knowledge, measured via 10 multiple choice questions; between-group difference in mean percentage correct was evaluated using 5,000 bootstrapped samples. Student attitudes on FND, serving as exploratory outcomes, were measured using Likert-style items; between-group differences were evaluated using standardized mean differences (SMDs), with |0.2|, |0.5|, and |0.8| interpreted as small, medium, and large effect sizes, respectively. Student feedback was also evaluated to guide future implementation.

**Results:**

Primary outcome data were available for 26/52 intervention site and 15/49 control site students. Intervention site students correctly answered more FND knowledge items (mean = 92%, SD = 8%) than students at the control site (mean = 72%, SD = 27%, mean difference = 20%, 95% CI 8.5%–36%). On FND attitude items, intervention site students demonstrated moderate effects in the direction of greater self-reported understanding (SMD = 0.57, 95% CI –0.11 to 1.3) and comfort with FND (SMD = 0.64, 95% CI –0.05 to 1.3). They also reported greater disagreement with the statement that psychiatrists, not neurologists, should care for patients with FND (SMD = −1.1, 95% CI −1.8 to −0.35). In their feedback, students valued learning about FND but questioned the need for 2 sessions.

**Discussion:**

Compared with a control group, medical students at a clerkship site that implemented case-based and near-peer didactic sessions demonstrated greater clinical knowledge of FND, as well as signals of more positive and less negative attitudes toward patients with FND. Although limited by sample size and response rates, these findings support the implementation of focused didactics on FND in neurology clerkships.

## Introduction

Functional neurologic disorders (FND) are highly prevalent^1^ and frequently identified among patients referred for outpatient neurology evaluation,^2-5^ with functional or psychological symptoms representing the underlying etiology for 16% of new patient presentations in a national study of neurology clinics.^5^ These disorders, which include conditions such as functional movement disorders and functional/dissociative seizures, significantly affect patients through decreased mental wellbeing^6^ and quality of life^7^ and generate large costs for health systems.^8^ Historically, FND have been defined negatively as neurologic symptoms without a localizable lesion.^9^ More recent work, however, has defined FND positively as diseases of network dysfunction^9^ and located FND among other disorders of brain-mind-body interface present across medical specialties and organ systems.^10^

Shortcomings in care for patients with FND, such as misdiagnosis and inappropriate treatment, may be common,^11^ and clinical education has been proposed as both a cause and cure for these deficiencies.^12,13^ Medical students^14^ and practicing clinicians,^15,16^ for example, consistently report insufficient training or understanding of FND, indicating a need for more dedicated curricula. Misconceptions and negative attitudes about patients with FND are also common among clinicians,^15-18^ transmitted to medical students during neurology clerkships,^13^ and reflected in the care provided to patients,^11,19^ highlighting a need for educational content that directly engages with these beliefs. Although educational programs on FND have previously been developed, they have focused on graduate medical education,^20^ suggesting opportunities for intervention earlier in medical training.

In response to these needs, we developed a multicomponent educational program on FND for medical students completing their core neurology clerkship. Given frequently reported feelings of helplessness and dissatisfaction among medical students caring for patients with FND,^13^ curricula were developed based on the framework of self-determination theory and implemented with the intention of fostering student experiences of autonomy, competence, and relatedness through case-based learning and small group reflection.^21^ Given the literature documenting deficits in both FND knowledge and clinician attitudes toward patients with FND, intervention effects were assessed in both of these domains and compared among students at intervention vs nonintervention clerkship sites. Clerkship feedback was also reviewed at study completion with the goal of refining future iterations of the program.

## Methods

### Sample

Participants in this study were medical students enrolled at Harvard Medical School who rotated through a mandatory, 4-week-long core neurology clerkship at either Beth Israel Deaconess Medical Center (intervention site) or Massachusetts General Hospital (control site) between January 2025 and December 2025. Students were made aware of random assignments to clinical sites 1 month before the start of medical school, approximately 18 months before the start of clerkship rotations. Among the entire class, 12 students opted to complete all core rotations at a community hospital (Cambridge Health Alliance) and were excluded from random clerkship assignment; all other students were randomly assigned in equal numbers to complete all core clinical rotations at Beth Israel Deaconess Medical Center, Massachusetts General Hospital, or Brigham and Women's Hospital, regardless of preference, academic performance, or any other factors, as documented in the medical school handbook.^22^ Students assigned to Cambridge Health Alliance or Brigham and Women's Hospital were not included in this study. Students were unable to take clinical electives at other hospital sites until completion of their core clinical rotations at their assigned site, precluding crossover at the time of the study.

### Intervention

Our educational program consisted of 2 hour-long didactic sessions delivered each month only at Beth Israel Deaconess Medical Center, the intervention site. The first session was led in-person by an attending neurologist during the second week of the clerkship. This session used a conversational, small group case-based learning approach aimed to elicit active participation from students. The case was based on a real patient's experience, with identifying information including name and age changed (available in supplementary materials). Predicated upon the conceptual framework of deliberate practice, there were several opportunities during the case for students to simulate how they would introduce the diagnosis of FND to the patient and counsel the patient regarding next steps in management. They received immediate feedback on these skills. Case content focused on a functional movement disorder that presented following a syncopal episode The core features of FND, internal inconsistency and incongruity, were discussed, and the pathophysiology and treatment of FND were also reviewed.

The second session took place during the final week of the month and was led by a medical student who had previously completed the neurology clerkship. This session began with an introduction locating FND within the broader diagnostic category of brain-mind-body interface disorders^10^ and discussed differences between FND and conditions that are frequently confused with FND (i.e., somatic symptom disorder, health anxiety, malingering, factitious disorder). Students were then asked to share their experiences caring for patients with FND and reflect on the interpersonal dynamics they observed and emotions that arose in the care of these patients. The facilitator would then share a brief TikTok video of a patient with FND discussing her positive experience with diagnosis and treatment.^23^ Following this, the facilitator would briefly discuss the history of myalgic encephalomyelitis/chronic fatigue syndrome, a condition associated with a decades-long debate regarding its etiology as either of immunologic or psychiatric origin, to highlight how conceptualizations of disease may reflect both available scientific knowledge and the societal context in which they exist. To conclude the session, the facilitator would provide strategies for advancing patient care and navigating difficult team dynamics when caring for patients with FND and other disorders of brain-mind-body interface in a medical student role.

Numerous aspects of these sessions align with the framework of self-determination theory, which posits that optimal growth for individuals comes when needs for autonomy (a sense of agency), competence (a sense of mastery), and relatedness (a sense of belonging) are met.^21^ For example, providing tangible tools designed to empower students in their specific clinical role aligned with needs for autonomy. Using a case-based format with clinical challenge moderated to meet medical student level aimed to provide students with a sense of competence. The use of reflective practice in a group of peers, modeled after Balint groups, aimed to meet need for relatedness.

Administrative support necessary for the intervention involved confirming availability of the facilitators each month and emailing calendar invitations to students and facilitators. The need to reschedule sessions was rare. A conference room located in the neurology department office was booked on a recurring basis and used to house the intervention sessions as well as other clerkship didactics. Students were informed of the expectation to attend all clerkship didactics at rotation orientation and given the noon hour free of clinical duties to participate. Following the session led by the medical student, the faculty facilitator would conduct a brief text message check-in with the student facilitator and send out the survey. Overall administrative burden spread between clerkship administrators and faculty was estimated to take approximately half an hour each month.

### Course Learning Objectives

1. Differentiate between syncope, epileptic seizures, and functional movements (functional/dissociative seizures, functional movement disorder, etc.) using key historical features (prodrome, recovery, incontinence, tongue bite) and initial evaluation (phenomenology of the movements).
2. Perform and interpret bedside examination maneuvers used to make the diagnosis of a functional neurologic disorder, with emphasis on the importance of internal inconsistency and incongruity in establishing the diagnosis.
3. Discuss how these positive clinical signs allow the examiner to rule in the diagnosis and review that FND is not a diagnosis of exclusion.
4. Explain the biopsychosocial model of FND by applying the 3 Ps framework: predisposing, precipitating, and perpetuating factors.
5. Describe the brain's “agency network” (including regions such as the temporoparietal junction) and explain how abnormalities in prediction and sensory integration within this network may lead to involuntary movements. Within this context, review the concept of top-down prediction error and impaired integration of bottom-up sensory inputs.
6. Distinguish FND from feigning or malingering and identify common myths and stigmatizing beliefs (e.g., “it's all in your head,” “patients are faking”) that pertain to the care of patients with FND. Review nonstigmatizing language to explain why symptoms are real, involuntary, and potentially reversible.
7. Develop and practice a patient-centered strategy for communicating the diagnosis of FND, including validating symptoms and explaining “functional” vs “structural” disease.
8. Outline an initial management plan for FND that includes patient education (discussing how and why functional symptoms may develop), targeted physical and psychological therapies, treatment of comorbid conditions (such as underlying anxiety or post-traumatic stress disorder), and the importance of close follow-up.
9. Examine FND and other disorders of brain-mind-body interface from a historical perspective to critically engage with common stigmatizing beliefs and unpack personal experiences providing patient care.

### Control

Students assigned to complete the neurology clerkship at Massachusetts General Hospital served as the control group. These students did not receive any formal didactic sessions regarding FND during the study period. Students at both sites had roles and responsibilities typical for a medical student at a quaternary academic medical center, which may or may not have involved the care of patients with FND. While Massachusetts General Hospital houses a dedicated FND treatment program, students were not scheduled to rotate on this service during the study period.

### Outcomes

Anonymous, uncompensated surveys were sent to students at both clinical sites in the final week of the clerkship following completion of the curriculum at the intervention site. Given the significant volume of requests for participation in educational research and assessments that students receive, we also opted against collecting any pre-intervention data in an attempt to minimize “survey fatigue” and maximize the quantity and quality of post-intervention responses.^24^

Knowledge of FND, serving as primary outcome, was measured via 10 multiple choice questions written in the style of United States Medical Licensing Examination Step 2 and developed using the National Board of Medical Examiners item writing guide (items and answer key presented in the supplementary materials). All items had 5 responses with 1 correct answer; not all correct answer choices related to the diagnosis/treatment of FND (in several cases, the correct answer involved further workup of possible stroke or epileptic seizure). Items were written to align with a previously published list of 10 “myths” about FND^18^ and were based on real-world cases. Two attending neurologists with significant experience caring for patients with FND (AH and JZ) iteratively wrote the questions, with 1 drafting the items and the other providing peer review. Questions were not piloted before use in this study. Students were not automatically provided with an answer key after completion, but this was freely provided to any student who requested it.

Attitudes on FND, serving as exploratory outcomes, were measured using 7 individual Likert-style items on a 5-point scale (1—strongly disagree, 2—somewhat disagree, 3—neither agree/disagree, 4—somewhat agree, and 5—strongly agree). These were based on previously published survey data involving neurology clerkship students from a peer institution.^25^

Anonymous qualitative feedback from students on the clerkship as a whole, collected as part of routine clerkship assessment separate from this study, was also reviewed at the time of study completion to identify comments regarding the FND curriculum. We specifically examined comments from 2 free-response items regarding lectures that were helpful and lectures that could use improvement. Starting in September 2025, students were also asked to list 3 major strengths and 3 major weaknesses of the clerkship and we examined these items as well. Students were not asked to specifically comment on the FND curriculum. We did not conduct a formal thematic analysis, but aimed to evaluate comments to identify areas of success and opportunities for improvement in the curriculum.

### Covariates

Given the small size of each clerkship group (typically 3–5 students per rotation site), we did not collect demographic data to fully maintain anonymity. To understand the extent of participants' relevant prior clinical experiences, students were asked to respond to the following multiple choice item at the end of the survey: “the estimated number of patients I have seen with medically unexplained symptoms, such as FND, since starting medical school is about __” with options of 1–5 patients, 6–10 patients, or >10 patients. The term “medically unexplained” and contextualization of FND into this category was utilized given historic use of this language in the literature.^2,3^ We acknowledge, however, that this term inherently fails to reflect advances in understanding of the pathophysiology of FND and other brain-mind-body interface disorders and that its use can thus perpetuate stigma around these conditions through demedicalization.^10,26^ The use of this language in the manuscript is done solely for the purpose of accurately representing the item stem and corresponding participant responses.

### Analysis

Patient exposure was reported in the above categories using frequencies and percentages, with between-group differences evaluated using Fisher's exact test.

FND knowledge was summarized as the mean percentage correct across all completed items. Responses with less than 3 of 10 items completed were excluded. Measure reliability was assessed using Cronbach alpha (with ≥0.7 as a cut-off for acceptable internal consistency^27^) and by evaluating the percentage correct for each individual item (with a target of approximately 70% given that all items each had 5 answer choices^28^). Between-group difference in mean percentage correct with 95% CIs was then estimated using 5,000 bootstrapped samples to accommodate a non-normal distribution.

FND attitudes were summarized using mean and SD for each item and compared between groups using standardized mean differences (SMD) and 95% CIs. Point estimates of |0.2|, |0.5|, and |0.8| were interpreted to represent small, medium, and large effect sizes, respectively.^29^ No dichotomization of results were performed. The results were also reported graphically using bar charts to demonstrate the distribution of item responses, stratified by intervention vs control group.

### Standard Protocol Approvals, Registrations, and Patient Consents

This project was determined to be human subjects research exempt from institutional review board review. Owing to this study's status as educational research, we did not obtain formal consents from participants. Participation in all assessments was voluntary and anonymous.

### Data Availability

All quantitative data from knowledge and attitude assessments as well as statistical code used for analysis will be made available by request from any qualified investigator.

## Results

Fifty-two and 49 students completed the neurology clerkship at the intervention and control sites, respectively. Thirty-two of 52 (62%) students at the intervention site and 22/49 (45%) students at the control site began the survey; among them, 26/32 intervention site students and 15/22 control site students completed at least 3 items in the primary outcome measure and were included in this analysis.

Patient exposure was prevalent and similar between groups. Respective students at intervention and control sites who completed this item most commonly reported exposure to 1–5 patients (17/25, 68% vs 8/13, 62%), followed by 6–10 patients (6/25, 24% vs 5/13, 38%) and >10 patients (2/25, 8% vs 0/13, 0%, *p* = 0.520).

Items assessing the primary outcome of FND knowledge displayed acceptable internal consistency (Cronbach alpha = 0.71, 95% CI 0.60–0.82). Individual item difficulty ranged from 82% to 97% correct, with the exception of 1 item which was 49% correct, all diverging from the target difficulty of 70%. Item completion was similar between groups (intervention mean = 9.8/10 items completed, control mean = 9.4/10 items completed).

Students assigned to the intervention site answered more items correctly (mean = 92%, SD = 8%) than those at the control site (mean = 72%, SD = 27%, mean difference = 20%, 95% CI 8.5%–36%) (Table).

**Table:** Differences in FND Knowledge and Attitudes Among Students Assigned to Intervention vs Control Sites

	BIDMC (intervention), N = 26^a^	MGH (control), N = 15^a^	Difference	95% CI
Graded items^b^				
Percentage correct	92% (8%)	72% (27%)	20%	8.5%–36%
Likert-style items^c^				
I understand the concept of functional neurologic disorder	4.6 (0.6)	4.2 (0.6)	0.57	−0.11 to 1.3
I am comfortable discussing functional neurologic disorders with patients	4.0 (0.8)	3.2 (1.5)	0.64	−0.05 to 1.3
Based on history and exam, I can usually determine a patient with a true functional neurologic disorder from someone who is malingering (“faking it”)	3.2 (1.0)	3.2 (1.2)	−0.03	−0.70 to 0.64
Patients with a functional neurologic disorder have a psychiatric diagnosis underlying their symptoms	2.4 (1.0)	2.2 (0.6)	0.16	−0.51 to 0.83
Seeing patients with functional disorders negatively affects my interest in medicine	1.8 (1.1)	2.2 (1.4)	−0.32	−0.99 to 0.35
I get frustrated by patients with functional neurologic disorders	2.2 (1.2)	2.0 (1.2)	0.14	−0.53 to 0.81
Functional neurologic disorders should be treated by a psychiatrist rather than a neurologist	2.2 (1.0)	3.0 (0.6)	−1.1	−1.8 to −0.35

Abbreviations: BIDMC = Beth Israel Deaconess Medical Center; MGH = Massachusetts General Hospital.

a Mean (SD).

b Estimate = difference between groups calculated with 5,000 bootstrap samples.

c Scale where 1 = “strongly disagree” and 5 = “strongly agree”; BIDMC n = 25, MGH n = 13 for these items; estimate = standardized mean difference.

Students at the intervention site more often disagreed with the statement “functional neurologic disorders should be treated by a psychiatrist rather than a neurologist” compared with students at the control site (mean = 2.2, SD = 1 vs mean = 3.0, SD = 0.6, SMD -1.1, 95% CI −1.8 to −0.35) (Table, also eFigure 1 for distribution of item responses). Students at the intervention site also demonstrated numerically greater agreement with the statements “I understand the concept of functional neurologic disorder” (mean = 4.6, SD = 0.6 vs mean = 4.2, SD = 0.6, SMD = 0.57, 95% CI –0.11 to 1.3) and “I am comfortable discussing FND with patients” (mean = 4.0, SD = 0.8 vs mean = 3.2, SD = 1.5, SMD = 0.64, 95% CI –0.05 to 1.3); point estimates were of moderate effect size though confidence intervals included zero. In addition, students at the intervention site demonstrated numerically less agreement with the statement “seeing patients with FND negatively affects my interest in medicine” (mean = 1.8, SD = 1.1 vs mean = 2.2, SD = 1.4, SMD = −0.32, 95% CI –0.99 to 0.35), although effect size was small and imprecise. Ratings for other items were similar between groups.

Among free-response clerkship feedback data, we identified 11 total comments regarding the FND curriculum. Five of the 11 comments were positive. Representative examples included “FND lectures were very helpful because it's a subject matter which we are less exposed to. “Another wrote” and the FND session was a nice addition in my opinion! Might be interesting/helpful to add something similar to other services for their versions of ‘functional’ disorders (like irritable bowel syndrome in IM/Surgery or what not). “The remaining 6 comments expressed that 2 sessions were too many or were redundant. Multiple comments expressed concern with how the curriculum aligned with shelf preparation, with 1 student writing while I appreciated the focus on FND given its high prevalence in all specialties, I think 1 of the sessions could have been better used to focus on subjects more heavily tested on the shelf.”

## Discussion

In this educational trial, medical students who received a didactic program demonstrated greater clinical knowledge of FND as well as signals of more positive and less negative attitudes towards patients with FND. These findings align with other curricula implemented on the graduate medical education level that produced similar gains in self-reported knowledge and attitudes on FND.^20^ Given the pressing need for increased education on FND, as well as the feasibility and effectiveness of this program, we believe these results support the broader implementation of dedicated curricula on FND into undergraduate medical education.

Although differences in knowledge observed in this study were substantial, differences in attitudes were modest and for the most part had confidence intervals that included the possibility of null or negative effects. Student attitudes on FND have been hypothesized to be the product of multiple components, including the interpretation of personal emotional experiences and role modeling from near-peers such as residents and attending physicians.^13^ While this intervention aimed to address both of these components, we were struck by the extent to which many students reported being emotionally impacted by caring for patients with FND. Some students, for example, noted trepidation about caring for future patients with FND after previous challenging experiences. Others disclosed unresolved moral distress. As educators and advocates aim to change clinician attitudes on FND, both our formally collected data and experience during implementation suggest that identifying methods to meaningfully discuss and reframe difficult experiences with patients and team members may be crucial to this goal.

Our findings of limited intervention effects on attitudes may also reflect the extent to which beliefs about FND are ingrained in institutional cultures. While our intervention operated on the level of individual learners, future interventions aiming to change beliefs about FND may need to move upstream and target larger communities (e.g., neurology departments, medical school curricula, hospital staff) to affect individual attitudes by changing culture. Although not intended, we are optimistic that conducting this project may have been a spark for broader cultural change in our own academic community; after participating in this trial, the control site launched its own monthly didactic for students on FND.

Clerkship feedback also highlighted the context of competing demands that students and educators exist in. While some students valued the program, many felt that 2 sessions was excessive or misaligned with other needs, such as preparation for the shelf examination. The current iteration of the program now only includes 1 session led by the neurology faculty member, a change made in response to learner feedback that the 2 sessions were redundant. We felt comfortable making this change given that large intervention effects on student knowledge suggested room for dose de-escalation, and limited impact on attitudes suggested that alternative approaches could be warranted. From an administrative perspective, while not explicitly tracked in this study as an outcome, time spent on logistics and delivery of the intervention was relatively minor and additional duties were able to be accommodated effectively by faculty and administrators involved, providing a general picture of intervention feasibility.

A novel feature of this educational intervention was the contextualization of FND into the larger diagnostic category of disorders of brain-mind-body interface,^10^ done with the intention of establishing a lens that students can apply across all specialty rotations. Future opportunities for research include expanding this approach to other “invisible illnesses” under the umbrella of disorders of brain-mind-body interface (i.e., chronic pain or disorders of gut-brain interaction in a primary care clerkship). Other avenues for research include examining the program's longer-term impact on attitudes and conducting “dismantling” research to determine which elements of this multicomponent intervention were responsible for the observed effects.^30^

Our findings have limitations. Response rates were low, although were higher than expected for a voluntary, uncompensated survey, which limited sample size and thus estimate precision. Response rates differed between groups, with fewer responses in the control group; we suspect that students in the control group who completed the survey may have had greater interest in FND, however, which could have potentially biased results towards the null. We also developed our own outcome measure to objectively measure student knowledge and align with stage 2 of evaluation in the Kirkpatrick model^31^; many items displayed insufficient difficulty, which could have similarly led to disproportionally inflated results in the control group and biased results to the null. Given prior research demonstrating that simply participating in neurology clerkships increases FND knowledge,^25^ relatively high scores on this outcome among both groups were expected, but future interventions may benefit from piloting assessments to ensure adequate item difficulty. Finally, the control site institution houses a dedicated FND treatment program and the intervention site does not, which could have potentially affected the faculty and patients whom students interacted with; clerkship students did not rotate through this program however, and reported clinical exposure was similar between groups.

In conclusion, this theory-informed curriculum on FND improved students' knowledge and demonstrated signs of reducing stigmatizing attitudes toward patients with FND. Broader implementation of such curricula may hold promise as a strategy for closing gaps in the care of patients of FND.

## Glossary

FND: functional neurologic disorder
SMD: standardized mean difference
